# Evaluation of the metastatic potential of malignant cells by image processing of digital holographic microscopy data

**DOI:** 10.1002/2211-5463.12282

**Published:** 2017-09-02

**Authors:** Violeta L. Calin, Mona Mihailescu, Eugen I. Scarlat, Alexandra V. Baluta, Daniel Calin, Eugenia Kovacs, Tudor Savopol, Mihaela G. Moisescu

**Affiliations:** ^1^ Biophysics and Cellular Biotechnology Department Faculty of Medicine Carol Davila University of Medicine and Pharmacy Bucharest Romania; ^2^ Physics Department Faculty of Applied Sciences Politehnica University of Bucharest Romania; ^3^ Applied Electronics and Informatics Engineering Department Faculty of Electronics Telecommunications and Information Technology Politehnica University of Bucharest Romania

**Keywords:** bimodality coefficient analysis of quantitative phase image, clonogenic test, digital holographic microscopy, impedance‐based cell proliferation test, metastatic potential, murine B16 malignant cells

## Abstract

The cell refractive index has been proposed as a putative cancer biomarker of great potential, being correlated with cell content and morphology, cell division rate and membrane permeability. We used digital holographic microscopy to compare the refractive index and dry mass density of two B16 murine melanoma sublines of different metastatic potential. Using statistical methods, the distribution of phase shifts within the reconstructed quantitative phase images was analyzed by the method of bimodality coefficients. The observed correlation of refractive index, dry mass density and bimodality profile with the metastatic potential of the cells was validated by real time impedance‐based assay and clonogenic tests. We suggest that the refractive index and bimodality analysis of quantitative phase image histograms could be developed as optical biomarkers useful in label‐free detection and quantitative evaluation of cell metastatic potential.

**Abbreviations**
CI: cell index
DHM: digital holographic microscopy
QPI: quantitative phase image
RI: refractive index
RTCA: real time cell analysis

With cancer incidence rates increasing, early and accurate diagnosis of malignancy is a major issue in biomedical research. Malignant cells have well‐known features that include cell cycle distortions, increased proliferation, anchorage independence and lack of contact inhibition of their growth. Advanced stages of malignancy are characterized by the ability to invade normal tissues and produce metastasis 1. Increased nuclear and cellular size, high DNA content and irregularities in chromatin structure are also observed 2.

Electrical, mechanical, optical and other biophysical techniques 3, 4, 5 have been used in the past decades to identify cell characteristics that could indicate malignancy. The cell refractive index (RI), for instance, was shown to be an optical parameter with high biological significance, being related to cell content and morphology 6. Recent advances in microscopy (especially confocal, scattering and interference microscopy) allow single‐cell measurement of the RI 7, 8. Non‐invasive measurements of the RI have been made also with infrared refractometry 9, optical cavity resonance 10, optical trapping 11 and real time optical plasmon resonance 12.

Various quantitative phase microscopy techniques such as digital holographic microscopy (DHM) have been used for biological sample imaging, using the optical path delay as an endogenous ‘contrast agent’ 13. As the observed phase shifts are determined by both the refractive index and cell height, several methods have been established to determine each of them individually 14, 15, 16.

Recent advances have made possible refractive index mapping within the whole cell volume by optical tomography 17, 18. As the RI is related to the cell density, dry and wet cell content and cell metabolism, a series of RI‐based biological applications have been proposed. Among these are monitoring transmembrane water fluxes 19, 20, determining ionic channel activity 21, 22, studying the impact of various chemical and physical agents on the cell cytoskeleton 23, detecting apoptosis 24, exploring cell dynamics 25, 26 and finding biomarkers for cancer and infectious and genetic diseases 27, 28, 29.

In the field of cancer pathology, it has been reported that the RI measured for individual cells is higher in malignant than in normal cells; this characteristic was attributed to the higher protein content of cancer cells sustaining a fast cell division 10, 17, 30, 31, 32, 33, 34. It was shown that even ‘uninvolved cells’ (histopathologically normal cells identified in tumors collected from cancer patients) have an elevated RI 35, 36. The RI measurement could be made on either adherent or suspended cells 37, 38.

Depending on the nature of the biological sample (e.g. attached live cells, fixed biopsy slices), the relationship between the RI and malignancy may be complex. Wang *et al*. found no differences in the RI of cancer and normal cells in unstained biopsies, but significant differences in RI spatial distribution in the slice 39. They proposed this distribution as a cancer biomarker. Giannios *et al*. (40) measured the RI on freshly excised human intestinal specimens; they found, in some patients, RI values of malignant mucosa tissues to be lower than in normal tissue 40. The authors attributed this difference to the extracellular matrix modification and fluid accumulation due to inflammation, apoptosis and necrosis.

When analyzing those results that seem to be contradictory, we have to keep in mind that measurement of the RI is strongly dependent on the experimental condition (live or fixed cells, single cell *versus* tissue, temperature, osmolarity) and on the resolution of the method (effective RI or 3D RI map) 30.

Apart from the RI and cell height, other cell parameters were defined based on reconstructed quantitative phase images (QPIs): dry mass, dry mass density and such shape‐related characteristics as eccentricity and sphericity indices. It was thus possible to monitor the cell cycle and cell growth, based on the phase profile parameters 41, 42.

Statistical analysis of the phase shift distribution within QPIs may be used to differentiate between normal and malignant cells: opto‐mechanical characteristics of malignant cells were investigated 43 and circulating tumor cells were isolated and monitored 44. Fingerprints of tumor cells were introduced by *in line* DHM, based on scattered light intensity and cell size 45.

Another statistical approach is the bimodality analysis of the frequency distribution of a parameter (already used in economics, psychology, agriculture and medicine), which characterizes the population heterogeneity and reveals the presence of hidden subpopulations 46. Bimodality analysis of breast tumor proliferative activity was correlated to prediction of the overall survival rate 47. Bimodality of blood glucose distribution was also used to identify a subpopulation with high prevalence of diabetes and obesity 48.

Here, we employed an *off‐axis* DHM method to reveal differences between two sublines (F1 and F10) of murine melanoma B16 cells, characterized by different metastatic potential. We computed the RIs of adherent cells in specific zones and characterized the phase shift distributions of the reconstructed QPIs of cells using the bimodality coefficient. Dry mass density of both sublines was also computed. The observed correlations of the RIs, dry mass density and QPI bimodality profiles with the cell metastatic potential were validated by two other methods that quantify cell proliferation rates, a clonogenic test and impedance‐based cell index recordings, which are standards for cell malignancy evaluation 49, 50, 51.

## Materials and methods

### Cells

The B16F1 and B16F10 sublines of B16 murine melanoma cells were kept in culture as recommended by the American Type Culture Collection (Manassas, VA, USA) at 5% CO_2_ and 37 °C (with a Heracell 150i incubator, Thermo Fisher Scientific, Waltham, MA, USA).

Cells were routinely cultured in 25 cm^2^ flasks (TPP, Trasadingen, Switzerland), using Dulbecco's modified Eagle's medium (DMEM) containing 4.5 g·L^−1^ d‐glucose, supplemented with 1 mm l‐glutamine and 10% fetal bovine serum (supplemented DMEM; cell culture components purchased from Sigma‐Aldrich, Steinheim, Germany). After detaching the cells with trypsin/EDTA solution (0.5 g·L^−1^ porcine trypsin, 0.2 g·L^−1^ EDTA·4Na in Hanks' balanced salt solution with phenol red; Thermo Fisher Scientific), the cells were counted (TC10™ Automated Cell Counter, Bio‐Rad, Hercules, CA, USA).

The B16F1 and B16F10 sublines had the same passage number (25) when the experiments began.

### DHM experiments, image acquisition and data processing

Cells were counted and seeded at (5–10) × 10^4^ cells·mL^−1^, on round glass microscope slides of 2 cm diameter, 24 h prior to the holography experiments. The slides with attached cells were mounted in a custom made manual perfusion chamber (Fig. 2A).

Holograms were recorded in an off‐axis experimental set‐up based on a Mach Zehnder interferometer, working in transmission 52, schematically presented and described in Fig. 2B. For the decoupling procedure, two holograms were acquired for the same cell bathed in two iso‐osmotic, neutral pH media having different refractive indices (Abbe refractometer, Euromex, Arnhem, the Netherlands): (a) DMEM without phenol red, with 1 g·L^−1^ d‐glucose and pyruvate (Thermo Fisher Scientific), RI = 1.3360 ± 0.0001; (b) 300 mm mannitol solution (Sigma‐Aldrich, Saint‐Quentin‐Fallavier, France), prepared using ultrapure water (18.2 MΩ·cm at 25 °C, Smart2Pure Ultrapure Water Systems, TKA, Niederelbert, Germany), RI = 1.3411 ± 0.0002.

Quantitative phase images were reconstructed using the dedicated commercial software koala (Lyncée Tec SA, Lausanne, Switzerland) following standard routines of the software 53. A QPI represents a phase map that associates to each pixel of the image a phase shift in degrees (Fig. 2D). Cell projections are delivered in the horizontal plane, and the phase shift introduced by the cell is represented on the vertical axis. The phase shift corresponds to the real optical path of the laser beam travelling through the cell, containing thus combined the cell RI and thickness information for each pixel of the cell image 54. For independent computation of these two parameters the decoupling procedure was applied, using two perfusion media with different refractive indices 15. Cell RI and height were computed on a square area of 3 × 3 pixels, identified by a matlab (Mathworks, Natick, MA, USA) code in the region of maximum phase shift, following the procedure described in our previous work 52.

Dry mass density of the cellular matter was computed from QPIs according to 19, as phase shift values in each pixel occupied by the cell, multiplied by the constant factor λ/2πα, where λ = 635 nm and is the laser wavelength, and α = 0.2 mL·g^−1^ and is the refractive increment of proteins 6. Average dry mass density values were computed for each cell.

Histograms of the phase shift value distribution within each cell image were built. Phase shift values were normalized as 8‐bit maps, attributing to the lowest phase shift value a pixel value of 0 and to the highest a pixel value of 255. Sarle's formula (Eq. (1)) for the multimodality coefficient *b* was used to characterize the differences between the F1 and F10 sublines 46:

(1) $$\frac{b=m_{3}^{2}+1}{m_{4}}$$

where *m*_3_ and *m*_4_ are the skewness and the kurtosis of the histograms, respectively. A perfectly flat distribution has *b* = 0.555. A coefficient *b* greater than 0.555 indicates multimodal distributions, the maximum *b* = 1 being obtained for a distribution with two distinct populations; a *b* value smaller than 0.555 indicates single‐peaked distribution with the theoretical minimum *b* = 0 for a single valued population.

### Impedance‐based cell index real time recordings and proliferation rate evaluation

Cells were counted, diluted, seeded at 5000 cells/well in 16‐well E‐Plates and incubated at 5% CO_2_, 37 °C. Cell proliferation rates were recorded using an xCELLigence^®^ DP real time cell analysis (RTCA) instrument (ACEA Biosciences, San Diego, CA, USA) equipped with real time cell analysis software v.2.0.0.1301. The cells adhering to a gold microelectrodes placed on the bottom of the culture well determine an impedance increase when low voltage alternating current is applied 55. The output of RTCA is the non‐dimensional parameter cell index (CI), which represents the difference between the measured impedance and the impedance without cells, normalized to the nominal impedance of the instrument. CI was recorded every 30 min during 5 days.

For each subline, the slope parameter was calculated over an interval between the recording time moment of 480 min (when the CIs of both sublines were similar: 0.4260 and 0.3993) and the moment of maximal CI value (6180 min, CI = 5.5562 for B16F1 cells and 5520 min, CI = 6.3842 for B16F10 cells).

### Clonogenic test and colony data processing

Cells were counted, diluted and seeded at 100 cells/dish in 6 cm diameter Petri dishes (TPP, Switzerland) using supplemented DMEM and incubated at 5% CO_2_, 37 °C for 2 weeks. The colonies were fixed with formaldehyde (diluted with H_2_O to 6%) (36.5–38% in H_2_O, Sigma‐Aldrich, Germany) and stained with crystal violet (0.5% in H_2_O) (Fisher Chemicals, Zurich, Switzerland).

**Figure 1:**
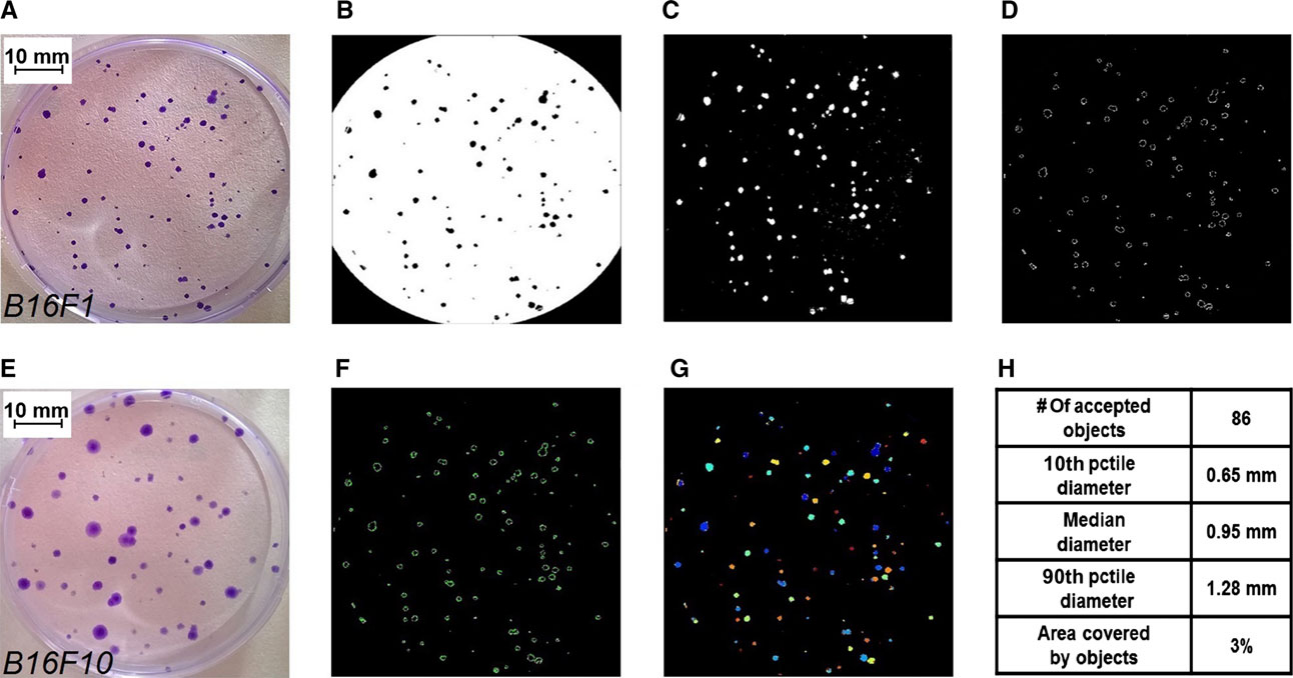
Images of cell colonies of sublines B16F1 (A) and B16F10 (E). One hundred cells were seeded per 6 cm diameter Petri dish and incubated at 5% CO_2_, 37 °C for 2 weeks; colonies were fixed with formaldehyde and stained with crystal violet. A schematic cellprofiler pipeline was created for measuring the number of colonies and the total area occupied by the colonies and is presented as an example for a B16F1 covered Petri dish shown in (A). After the Petri dish image was cropped to a circle and converted to an 8 bit grey image, it was converted to a black and white binary image (B) by applying a threshold filter. Then the image was inverted to white and black (C); the program identified the edges of all objects (D) and completed the incomplete edges by applying a Fourier transform (F). The colonies were identified as all complete‐edge objects (G) having a diameter in the range of 10–300 pixels. The output metadata consisted of number of colonies (# of accepted objects) and total area occupied by colonies (Area covered by objects) calculated as a percentage of the Petri dish area (H).

The differences between the sublines as number of colonies and average area of each colony (Fig. 1A,E) were evaluated by using the open‐source software cellprofiler v.2.1.1 39, 56. An image processing pipeline was developed by taking as entry one image or bulk images of the cell colonies (Fig. 1B–D,F–H). Briefly, the working modules allowing automatic extraction of the programmed output data were the following.

- Image processing modules: the images were cropped to a circle corresponding to the borders of the Petri dish, converted to grayscale (8 bit), then converted to a black and white binary image (Fig. 1B) by applying a threshold filter; images were subsequently inverted to white and black (Fig. 1C); the program identified the edges of all objects (Fig. 1D) then completed the contour for the objects with incomplete edges, by applying a Fourier transform (Fig. 1F).
- Object identification module: the objects were identified as all the completed edges (Fig. 1G).
- Measurement module: the program sorted all objects having the diameter in the range of 10–300 pixels; the area of all sorted objects was calculated using a numeric computation of all pixels inside the sorted object edges and divided by the initial cropped area; the output metadata consisted of the number of colonies and total area of colonies (as percentage of the Petri dish area; Fig. 1H).

### Statistics

Five independent DHM experiments were performed for each subline. Three experiments in duplicate were made for RTCA evaluation and in triplicate for clonogenic tests.

Results are presented as the mean ± SD. Statistical significance was analyzed using the nonparametric Mann–Whitney *U* test for two independent samples. Differences were considered significant at *P* < 0.05.

## Results

### Digital holographic microscopy

**Figure 2:**
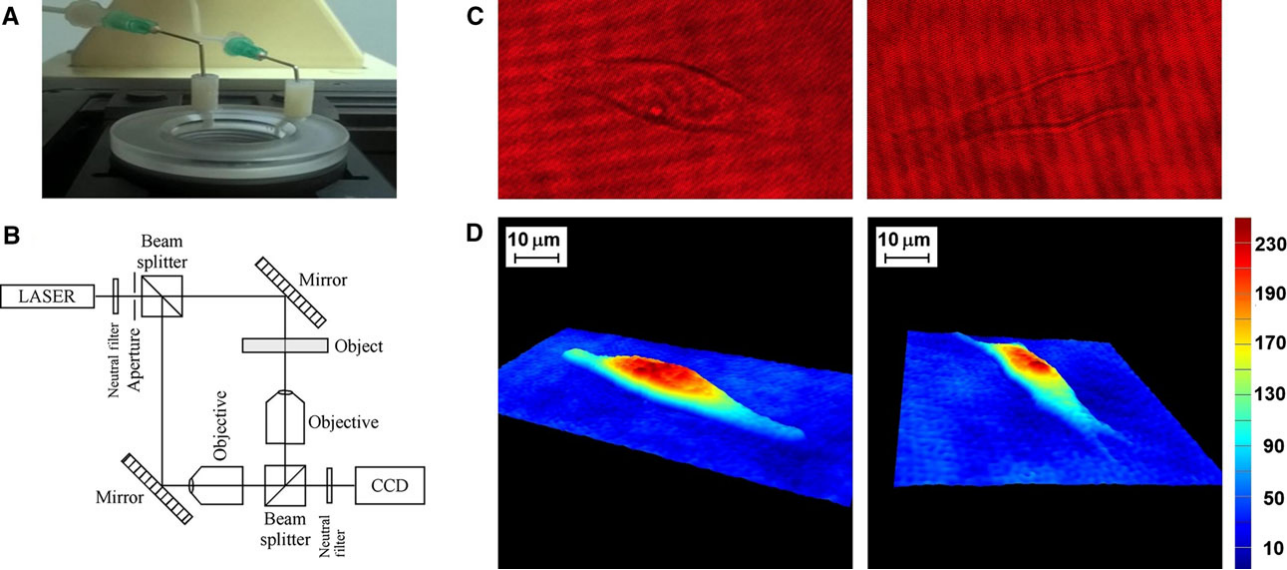
(A) Image of a custom made perfusion chamber containing 24 h cultured B16 cells. (B) Scheme of the digital holographic microscopy experimental set‐up based on the Mach–Zehnder interferometer, working in transmission. (C) Holograms of a B16F1 cell (left) and a B16F10 cell (right). (D) 3‐D quantitative phase images of the same B16F1 (left) and B16F10 (right) cells reconstructed using koala dedicated software 52.

Examples of holograms and quantitative phase images that were acquired and reconstructed for cell specimens from each B16 subline are presented in Fig. 2C,D. Using the decoupling procedure, the cellular RI and height were calculated in a square area of 3 × 3 pixels characterized by maximum phase shift in QPIs.

**Figure 3:**
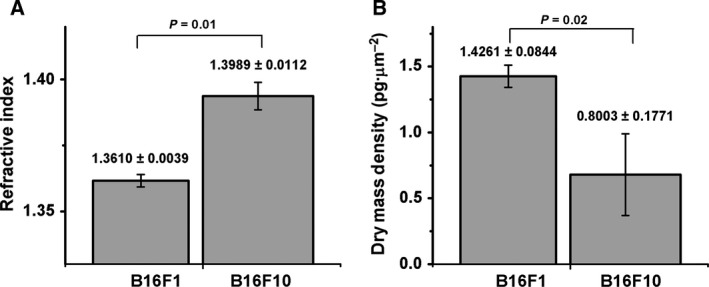
(A) Refractive Index of B16 cells calculated in an area of 3 × 3 pixels selected in the maximum phase shift zone of QPIs. (B) Dry mass density of B16 cells calculated using the phase shift value of each pixel multiplied by the constant factor λ/2πα (λ = 635 nm is laser wavelength, α = 0.2 mL·g^−1^ is refractive increment of proteins 19). Mean values ± SD were calculated for five cells for each subline.

We found that the refractive index of the F10 subline was significantly higher than that of F1 (*P* = 0.01) in the specified area (Fig. 3A). The average dry mass density of F1 cells had a significantly higher value compared with F10 cells (*P* = 0.02) (Fig. 3B). The cells' heights calculated in the maximum phase shift area were similar for both sublines, ranging between 4.60 and 11.14 μm (data not shown).

### Bimodality analysis

**Figure 4:**
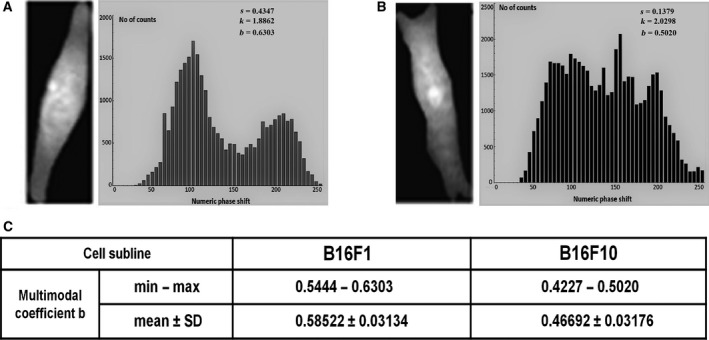
(A,B) Examples of quantitative phase shift maps and their corresponding histograms for B16F1 (A) and B16F10 (B) cells. The values of skewness (*s*), kurtosis (*k*) and multimodal coefficient (*b*) are presented. Histograms were computed based on the reconstructed QPIs (for the clarity the histograms are presented here with fewer bins on the abscissa; calculations were, however, performed using unitary resolution). (C) Statistics of multimodal coefficients for B16F1 and F16 F10 (five cells from each subline were used for calculations).

The distribution of the phase shifts within the whole cell area was different for the F1 and F10 sublines: in the case of F1 cells, the corresponding phase shift histograms exhibited a multimodal distribution with separated groups of dominant peaks (Fig. 4A) while the tendency to a single peaked distribution was charactersistic of F10 cells (Fig. 4B).

### Real time cell analysis

**Figure 5:**
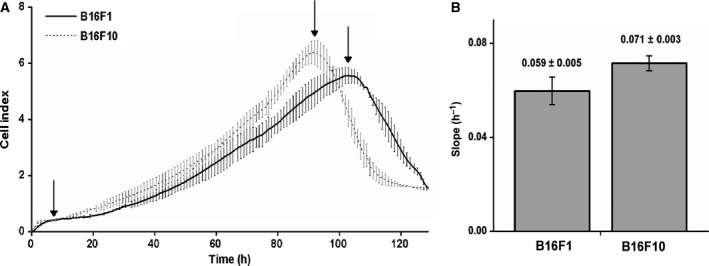
(A) Cell index was recorded for 5 days starting from the moment of cell seeding (5000 cells/well); the CI signal was measured every 30 min. (B) Slope parameter was computed for each subline; it was calculated between the recording time of 480 min (when the CIs of both sublines were similar: 0.4260 and 0.3993) and the recording time of maximal value (6180 min, CI = 5.5562 for B16F1 cells; and 5520 min, CI = 6.3842 for B16F10 cells); these times are indicated with arrows.

The 5 days' evolution of the cellular index of B16 cells is presented in Fig. 5A. The CIs recorded for the two sublines behaved quite similarly, reflecting cell adhesion, proliferation and detachment from the microelectrodes due to cell death. The evolution of the CI signal showed several phases. First, a rapid increase (1–2 h) of the signal occurred, caused by the cells coming to lie on the microelectrode array and by the normal adhesion process after seeding (cells were attaching, recovering from the stress of seeding and resuming their cell cycle). In a second phase, which lasted approximately 20 h, the signal increased at a slow rate (since the cell number was still low) corresponding to cell growth and incipient division. In a third phase, a faster increase of the CI signal occurred, generated by cell proliferation. Once the electrodes were fully covered by cells, the culture stopped growing, and the cells started dying and detaching from the bottom; this led to a rapid signal decrease in the last phase.

In spite of the comparable shape of CI curves of the two sublines, the maximum CI value was higher and was reached faster in the case of the F10 subline (as a result of the higher division capacity) as compared with F1. Moreover, in Fig. 5B, one can observe the higher slope values in the case of B16F10.

### Clonogenic test

**Figure 6:**
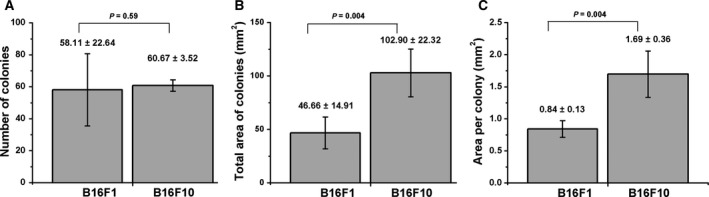
The ability of the two sublines to form colonies. Aliquots of 100 cells were plated in Petri dishes; after 2 weeks, the colonies were fixed and stained. The following parameters were computed using cellprofiler software: total number of colonies per plate (A) and total area of colonies per plate (B). The area per colony was computed (C).

Information about grown colonies is presented in Fig. 6. The counted number of colonies produced by the two sublines was similar, around 60 colonies per dish. The total area of colonies (and consequently the average area per colony) was, however, more than double in the F10 subline as compared with F1.

## Discussion

Measurements of the refractive index and size of living adherent cells were made using digital holographic microscopy in the off‐axis configuration, without using any exogenous contrast agents. The cell refractive index and the dry mass density were compared for two sublines of B16 murine melanoma cells of different metastatic potential. Further, using the bimodality analysis, we identified different characteristics of the phase shift distribution in the reconstructed quantitative phase images of the two sublines.

In our study we have used the B16F1 and B16F10 cell sublines, from the series selected by Isaac Fidler in 1973 57. The existence of the B16 murine melanoma sublines differentiated on the base of metastatic behavior offers a good *in vitro* model for studying the biophysical characteristics of malignant cells and their changes in the course of cell transformation. The selection of B16 variants was based on their increasing potency to form lung colonies after intravenous injection in mice. For this purpose, Fidler performed intravenous administration of B16 cells followed by subculturing of the induced pulmonary tumors in repetitive cycles 57. In this way, sublines with different metastatic behavior were differentiated, the metastatic potency increasing with the attributed index: B16F1 has the lowest potency to form pulmonary tumors while B16F10 has the highest. Fidler demonstrated also that selected sublines keep their metastatic properties after many passages 58. During the last 40 years, B16 variants have been characterized from morphological, biophysical and biochemical perspectives. B16F10 cells were characterized as being more adherent and having a higher tendency to aggregate (to adhere to each other or to other cell types) as compared with the F1 subline 59, 60. As concerns biochemical differences, higher levels of proteases and glycosidases were reported in F10 as compared with subline F1 61. B16F10 has a much lower cholesterol/phospholipid ratio than F1 62. Higher levels of the Sfrs1 protein, which promotes cancer growth and development of metastasis, were found in the cytoplasm and cell surface in the case of F10, as compared with F1; galactin‐3, associated with cell migration and invasion in melanoma, was also found in higher amounts in B16F10 cells 63.

Briles and Kornfeld found no difference in morphology and detachment properties between the B16F1 and B16F10 lines 64. As regards the cell morphology, in our work we found the cell height to be in the range 4.60–11.14 μm, similar for both sublines. The large range of height values may be explained by the heterogeneity of cell populations as they were in different stages of the cell cycle. A similar observation was made by Polo‐Prada *et al*. regarding the cell diameter: they found it to be 19.56 ± 6.57 and 20.79 ± 10.55 μm for B16F1 and B16F10 adherent cell sublines, respectively 65. They highlighted that cells coming from the same source exhibit height variability. This makes it difficult to identify a specific cell type, in view of cancer detection based only on morphological features.

As regards the optical properties, we found that, in the maximum phase shift area, F10 cells have a higher RI than F1. The maximum phase shift area has been associated, in many works, with the nucleus area, which is also the region of maximum cell height. The cellular RI is known to be influenced by the protein concentration, increasing by 0.002 units for each percentage of protein content 6. The RI was found to be heterogeneous within the cell and it is assumed that regions with high RIs correspond to organelles, but the RIs of each organelle are, however, still under study 30.

There is an abundant literature investigating the differences in the RI of cancer and normal cells. Choi *et al*. described that RI is higher in malignant cells, proposing the RI as a biomarker for cancer diagnosis 17. Using full‐field optical coherence microscopy, they reconstructed RI maps of adherent living cells for pairs of normal and cancerous cell lines from the same origin (normal kidney epithelial RK3E and k‐ras‐transformed RK3E rat cell lines, respectively normal immortalized oral keratinocytes INOK and oral squamous carcinoma YD‐10B human cell lines). Mean RI values were found to be 1.353 ± 0.008 for normal and 1.371 ± 0.014 for cancer cells. By using different cell types, it has been shown that irrespective of the cell type, RI is higher in malignant cells 10, 33. The RI difference was attributed to the high protein accumulation into the cell organelles (mainly the nucleus) due to the rapid cancer cell division and higher proliferation rate. Moreover, Bista *et al*. proposed the nuclear RI to be a measure of the nuclear mass density; they reported values of nuclear RI above 1.5496 that vary during the cell cycle caused by the variations in DNA content 32. Hartman *et al*. assessed the nanomorphology of various cells' nuclei from biopsies of pancreatic–biliary tumors, by measuring the average optical path delay; they showed that this parameter may be used to differentiate between cancer, uninvolved and normal cells 66. The differences were attributed to changes in nuclear density and spatial rearrangements in chromatin structure following the genetic mutations responsible for cancer transformation. Presently, RI mapping with high resolution can be performed, contributing to cancer risk evaluation 34.

The RI values found in our study (1.3610 ± 0.0039 for B16F1 and 1.3989 ± 0.0112 for B16F10) are in good agreement with the results summarized above. The higher RI values of F10 could be a potential tool to discriminate not only between normal and malignant cells, but also between malignant cells with different metastatic potential.

Moreover we found that the dry mass density, which characterizes the protein content of entire cells, is higher for F1 than for F10 cells (Fig. 3B). The mean values (1.4261 ± 0.0844 pg·μm^−2^ for F1 and 0.8003 ± 0.1771 pg·μm^−2^ for F10) are in agreement with those reported in studies where this parameter was followed up during the cell cycle 41, 67.

Dry mass density is correlated to the non‐aqueous content of the cell, quite similar to the classical density. Using gradient density separation technique, Baniyash *et al*., showed that in unselected B16 tumor cells there are cell populations with different densities 68. A lower density population proved to be more successful in lung colonization and was selected as F10, while a high density population had lower lung colonization capacity and was selected as F1. Our result confirms the observation that a higher metastatic capability is associated with a lower density; the dry mass density could thus act as an early biomarker for cell metastatic potential.

We looked for another optical biomarker for metastatic potency using the bimodality coefficient method to analyse the QPIs. Examining Fig. 4, it can be seen that the F1 cell histogram shows a bimodal distribution of the phase shift within the cells, while the F10 cell histogram shows a tendency to a single‐peaked distribution. Although we worked only on five cells belonging to each subline, the kurtosis and skewness parameters were computed for all pixels of each phase image providing a reliable amount of data, suitable for high order analysis. Degree of bimodality is an important feature of a frequency distribution, because it suggests irregularities, such as polarization or two underlying distributions combined into one. Sharp phase shifts, corresponding to the refractive indices of the compact nuclei, characterize normal cells, which differ from abnormal cells with irregularly enlarged nuclei 69, 70. Normal cells exhibit multimodal distributions with clearly separated groups of dominant peaks (nucleus and cytoplasm peaks) while the tendency to single peaked distribution might be a sign of abnormality.

Our results from RTCA and clonogenic tests confirm a higher proliferation capacity of the B16F10 relative to the B16F1 subline (higher CI and larger colonies as can be seen in Figs 5 and 6).

Our findings lead to the conclusion that QPI bimodality analysis combined with the refractive index and dry mass density measurements show promise for discriminating between cells with different metastatic potential.

## Conclusions

This work proposes new optical biomarkers to characterize cell metastatic potential: QPI bimodality analysis combined with refractive index and dry mass density computations. Our results show that the subline with higher metastatic potential presents a unimodal histogram signature, higher refractive index and lower dry mass density. Here we presented data to illustrate the principle of the approach, further developments (synchronized cells and automatic detection algorithms) being in progress. The possibility of extending the method from single cell analysis to a larger number of attached cells and even to tissues is to be considered. Measuring the phase shift by using a miniaturized portable device based on the interferometric technique and the subsequent data processing might have clinical potential, contributing to optimization of cancer medicine.

## Data accessibility

Research data pertaining to this article have been deposited at figshare.com https://dx.doi.org/10.6084/m9.figshare.5311108.

Digital holographic microscopy (DHM) images were acquired for two sublines (F1 and F10) of murine melanoma B16 cells, characterized by different metastatic potential. Reconstructed quantitative phase images (QPIs) were used to compute parameters such as refractive index (RI) and dry mass density or to obtain phase shift distribution within the cells.

**Fig**. 1 Images of colonies grown for B16 sublines and a schematic cellprofiler pipeline created for quantifying these colonies.

**Fig**. 2 DHM off‐axis set‐up, holograms and QPIs of the two B16 sublines.

**Fig**. 3 RI and dry mass density parameters.

**Fig**. 4 Histograms of phases from two QPIs of cells belonging to the two sublines, giving the values of the multimodal coefficient used to analyze the histograms.

**Fig**. 5 RTCA results.

**Fig**. 6 Colony analysis.

## Author contributions

MGM, MM and VLC conceived the study; MGM, TS and EK supervised the study; MGM, MM and TS designed the experiments; VLC and MM performed the experiments; MM, ES and AVB processed the DHM data; VLC processed RTCA data; DC processed data for clonogenic test using cellprofiler and prepared the figures for manuscript submission; VLC, MGM, MM and ES wrote the manuscript; MGM, EK and TS revised the manuscript; all authors approved the submitted manuscript.
